# Evolution and Phylogenetic Diversity of Yam Species (*Dioscorea* spp.): Implication for Conservation and Agricultural Practices

**DOI:** 10.1371/journal.pone.0145364

**Published:** 2015-12-21

**Authors:** Marie Florence Sandrine Ngo Ngwe, Denis Ndoumou Omokolo, Simon Joly

**Affiliations:** 1 Laboratory of Plant Physiology, Higher Teacher’s Training College, University of Yaoundé 1, P. O. Box 47, Yaounde, Cameroon; 2 Institut de recherche en biologie végétale, Montreal Botanical Garden and Université de Montréal, 4101 Sherbrooke East, Montréal, QC, H1X 2B2, Canada; 3 Institute of Agricultural Research for Development-CEREFEN, BP 167, Meyomessala, Cameroon; Saint Mary's University, CANADA

## Abstract

Yams (*Dioscorea* spp.) consist of approximately 600 species. Presently, these species are threatened by genetic erosion due to many factors such as pest attacks and farming practices. In parallel, complex taxonomic boundaries in this genus makes it more challenging to properly address the genetic diversity of yam and manage its germplasm. As a first step toward evaluating and preserving the genetic diversity yam species, we use a phylogenetic diversity (PD) approach that has the advantage to investigate phylogenetic relationships and test hypotheses of species monophyly while alleviating to the problem of ploidy variation within and among species. The Bayesian phylogenetic analysis of 62 accessions from 7 species from three regions of Cameroon showed that most *Dioscorea* sections were monophyletic, but species within sections were generally non-monophyletic. The wild species *D*. *praehensilis* and cultivated *D*. *cayenensis* were the species with the highest PD. At the opposite, *D*. *esculenta* has a low PD and future studies should focus on this species to properly address its conservation status. We also show that wild species show a stronger genetic structure than cultivated species, which potentially reflects the management of the yam germplasm by farmers. These findings show that phylogenetic diversity is a promising approach for an initial investigation of genetic diversity in a crop consisting of closely related species.

## Introduction

One of the main challenges of conservation biology is to cope with the ongoing global biodiversity crisis. The loss and fragmentation of natural habitats, pollution, invasive species, overexploitation of ecosystems and climate change are dramatically affecting biodiversity [1]. Species extinctions have the potential to decrease the ecological services offered by the ecosystems to humanity, but they also result in the loss of a singular genetic heritage constituted since speciation from their ancestral species. This is not only true of species, but also of taxonomic units below the species level [2]. Hence, local population extinction and genetic erosion due to fragmentation can have a profound influence on the loss of evolutionary uniqueness.

Evolutionary approaches are increasingly used in conservation because they help to identify species or regions at risk of extinction [3,4,5,6]. Moreover, they alleviate the problems associated with the lack of agreement on species concepts, which can affect standard biodiversity estimates [7,8]. One contribution of evolutionary biology to conservation biology is the concept of phylogenetic diversity (PD), which is defined by the sum of branch lengths of the evolutionary tree connecting a set of taxa or individuals [4]. Thus, a given set of taxa will have a greater PD if they are more spread out on a phylogenetic tree. The loss of PD is generally interpreted as a signal of declining biodiversity [9]. Moreover, PD is related to functional diversity [10] because evolutionarily distant species are more likely to have different functions in an ecosystem. Consequently, higher PD is also associated with more diverse eco-services [5, 11]. Evolutionary trees used for estimating phylogenetic diversity are also useful by themselves since species evolution is an important criterion in the conservation policy planning [12]. Phylogenetic hypotheses also improve our understanding of the current state of diversity and help to make predictions about the future [13].

*Dioscorea*, commonly called yam, is a tuber crop of great economic, social and cultural relevance in many tropical countries [14]. In Cameroon, yam is third after cassava and cocoyam/taro according to the volume of plant roots and tubers produced [15]. Usually consumed boiled, it contributes to food security in Africa. The genus *Dioscorea* comprises over 600 species [16]. Of these species, *Dioscorea cayenensis*, *D*. *rotundata* and *D*. *alata* are the most cultivated and of greatest economic interest in Africa [17]. In contrast to *D*. *cayenensis and D*. *rotundata* that are native to Africa, *D*. *alata* comes from Asia [18]. Despite their importance, yields are typically low in Africa due to pest attacks, diseases and their mode of propagation (vegetative multiplication). The vegetative propagation in yam is done from tubers or fragments tuber collected during the previous harvests. This mode of propagation favors the dissemination of pathogens in the field and prevents adaptation and the formation of new varieties. Indeed, the pest attacks and type of propagation are the main factors that contribute to genetic erosion of *Dioscorea* [19, 20]. This loss of diversity, which is manifested by the disappearance of local populations or varieties, lead to the loss of services that yams offer to humanity, especially in Africa. Hence, it is important to study the genetic diversity of species and understand their correlations to different environmental factors (climate, farming practices, etc.). A few studies have recently investigated the genetic diversity of several species in Benin, Nigeria, and Côte d’Ivoire [21, 22, 23]. Yet, similar efforts have been lacking in Cameroon, which is among the African countries with the greatest yam diversity with ten cultivated species, seventeen wild species and six species both cultivated and wild [23].

Assessing yam diversity is challenging for several reasons. One is that it is a taxonomically complex genus due to an important morphological diversity [19]. Yet, species is the fundamental unit of biodiversity and a good knowledge of species is important to preserve biodiversity. Another challenge is the important ploidy level variation observed within and across species. For instance, in Cameroon the level of ploidy varies from diploid in *D*. *dumetorum* to octoploid in *D*. *cayenensis* and some species have varying ploidy levels [24, 25]. This variation makes it hard to compare the level of genetic variation among populations or species using nuclear markers because an individual with a higher polyploidy level has more copies of each gene in its genome [26,27]. Hence, all else being equal, an octoploid species is expected to have greater genetic diversity than a diploid species. Moreover, because chromosome segregation patterns are poorly known, it is difficult to easily correct for this bias.

In this study, we use a phylogenetic diversity (PD) approach based on chloroplast DNA in order to assess genetic diversity within and across eight *Dioscorea* species of Cameroon. This approach has several advantages. First, unlike the nuclear genome that can be affected by ploidy variation, the chloroplast is always haploid and therefore investigation of the chloroplast genome would allow comparing the genetic variation of species irrespective of their ploidy levels. The chloroplast is suitable for such investigations as it is generally sufficiently variable to provide both inter-and intra-specific variations, especially when a highly variable region is selected [28, 29]. A phylogenetic diversity approach also has the advantage of allowing the reconstruction of phylogenetic relationships of species and as such it has the potential to enlighten potential taxonomic problems [30]. The markers *matK* and *rbcL*, the two plant barcode loci, have been used for phylogenetic studies of *Dioscorea* [31, 32]. However, these previous studies have established the phylogenetic relationships with only one specimen by species, which is not sufficient for testing species monophyly. Finally, it is much more easily applied across species compared to simple sequence repeats (SSR) that are often difficult to transfer between even closely related species. This is the case for *Dioscorea* where few SSR developed for *D*. *alata* could be transferred to its close relatives [33]. The objectives of this study are thus twofold: (i) to use a phylogenetic diversity approach as a proxy for estimating the genetic diversity of *Dioscorea* species, and (ii) to explore the phylogenetic relationships and test the monophyly of the *Dioscorea* species from Cameroon using several samples per species.

## Materials and Methods

### Plant material

**Fig 1:**
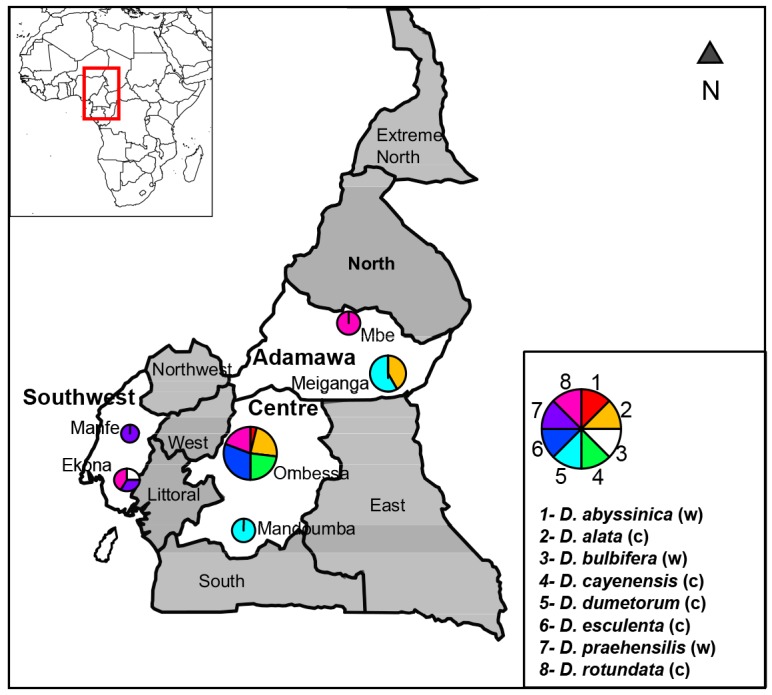
Map indicating collection sites for wild (w) and cultivated (c) of yam species. The sizes of circles are proportional to the number of accessions sampled, whereas the pie chart represents the relative proportion of each species at a given locality.

Sampling was conducted during the yams harvest period, in August and September 2011 and 2012. Tubers and plant specimens of five cultivated species (eleven accessions of *D*. *alata*, five of *D*. *cayenensis*, nine of *D*. *esculenta*, eleven of *D*. *dumetorum*, fifteen of *D*. *rotundata*,) were collected from farmer fields from the three main yam producing regions of Cameroon: Adamawa, Centre and Southwest. These collection sites were represented on a map generated with SimpleMappr [34] graphics with the R [35] software using the “maptools” [36] and “mapplots” [37] packages (Fig 1). In addition to the most important crop species mentioned in the introduction, *D*. *esculenta*, which might have originated in the Phillipines [38], and *D*. *dumetorum* were also included. Three wild species (one accession of *D*. *abyssinica*, three of *D*. *bulbifera*, seven of *D*. *praehensilis*) were collected in forests either close or far from the farmer fields. *Dioscorea bulbifera* is treated as a wild species here, but it is also sometimes cultivated [39]. It is distributed in pantropical regions [38] and is widely used in traditional Chinese medicine [40]. The other wild species studied, *D*. *abyssinica* and *D*. *praehensilis*, are important sources of diosgenin, a chemical used for the commercial synthesis of sex hormones, and corticosteroids that are widely used for antinflammatory, androgenic and contraceptive drugs [41, 42]. In total, 62 accessions were used in this study (Fig 1). Identifications of yam accessions were validated by the National herbarium of Cameroon. Harvested tubers collected were cultivated in an experimental field for ex situ conservation, whereas young leaves of each accession were immediately dried in silica gel for DNA extraction upon sampling in the field. Sampling was sometimes carried out on private land, in which cases permission from the owners was obtained prior to collecting. No permissions were required for sampling on public land.

### DNA extraction

DNA was isolated from 20–30 mg of dried leaves using the Plant extraction kit of BioBasic (Mississauga, ON, Canada). The purity and quantity of DNA extracts were checked by agarose gel (1%) electrophoresis and using a UV spectrophotometer (Thermo Scientific Nano drop, Montreal, Canada) at wavelengths of 260 and 280 nm.

### PCR amplification and sequencing

The *rbc*L and *rpl*32-*trnL* regions were amplified and sequenced on a subsample of eight accessions from all seven species to select the most variable region. The *rpl*32-*trnL* region, which was found to be the most variable (see results), was amplified and sequenced for all samples. Annealing temperatures followed the recommendations by Gao et al. [32] and Shaw et al. [28]. Polymerase chain reactions (PCRs) were carried out in 25 ul reaction mixture containing 1X DreamTaq buffer, 20 mM MgS02, 1U of DreamTaq DNA polymerase (Thermo Scientific), 0.3 μM of each dNTP, 50 μg BSA; 20 ng of DNA and 0.4 μM of rpl32f and TrnLr primer [28] or rbcLf and rbcLr. PVP ranging from 1 to 2% in concentration was added to the reaction mix to neutralize phenolic compounds capable of preventing the amplification of DNA [43]. PCR was performed on a thermocycler programmed for an initial denaturation step at 94°C for 3 min, followed by 40 cycles that consisted of 45 s at 94°C, 30 s at 58°C (for rbcL) or 50°C (rpl32), and 1 min at 72°C, and a final extension step at 72°C for 1min. Successful PCR products were sent for sequencing at the Genome Quebec Innovation Centre (Montréal, Quebec, Canada) and were sequenced using a 3730xl DNA Analyzer (Applied Biosystems, Burlington, ON, Canada).

### Marker comparison and selection

Sequences were edited, assembled and aligned using MUSCLE [44] in Geneious v 5.6 [45]. Primer-binding regions were removed from the alignments. Sequence characteristics, pairwise sequence divergence and parsimony statistics were calculated in PAUP v 4.0b10 using the Alltrees, Showdist and Showmatrix commands [46]. This allowed us to select the most variable and informative marker for the analysis of the whole dataset.

### Phylogenetic analysis

We conducted phylogenetic analyses for all taxa for *rpl*32-*trnL* region. *Dioscorea elephantipes* (NCBI accession number EF380353) was used as outgroup following previous phylogenies that suggested it is external to the studied species [31]. The best fitting substitution model for *rpl*32-*trnL* was selected using Akaike’s Information Criterion (AIC) with jModeltest2 [47]. Phylogenetic analysis was performed in a Bayesian framework using MrBayes v3.2.2 [48]. Two independent runs of four Monte Carlo Markov Chains (MCMC) were performed with the default temperature of 0.2 for the heated chains; a run length of 10,000,000 generations sampled every 1,000th, and a TPM1uf+G substitution model. A burn-in of 25% was removed from each run and the remaining 1,502 trees were combined using TreeCombiner [49] for the subsequent analyses. The program Tracer v1.5 [50] and the uncorrected potential scale reduction Factor (PSRF) in MrBayes were used to check for the adequacy of the burn-in and for the convergence of the Markov chains. The maximum clade credibility tree was obtained with TreeAnnotator and was visualized with FigTree v1.4.0 (http://tree.bio.ed.ac.uk/software/figtree/).

### Phylogenetic diversity and variation partitioning

The phylogenetic diversity was estimated using the PSV statistic [51] because it is independent of species richness, unlike the original PD statistic [52]. This is important in the present case because we had different sample sizes for the different species and regions. PSV was calculated for each species and for each region studied. Analyses of phylogenetic diversity were performed with the R [35] software using the ‘picante’ package [53].

To evaluate the importance of species assignment and regions in structuring the genetic variation observed in our data, we used variation partitioning [54]. Variation partitioning was performed through canonical redundancy analysis (RDA) in R using the ‘vegan’ package [55]. Adjusted *R* ^*2*^ values are reported and the significance of individual fractions was tested using partial RDA with 999 permutations, except for the shared fraction that is not testable.

## Results

### Marker comparison

Sequence divergence was higher for the *rpl32*-*trnl* spacer (0.0–5.0) than for the *rbcL* region (0.0–2.5) and the same trend was observed for the number of variable characters and parsimony informative characters (Table 1). The sequence alignment of the *rpl32*-*trnL* region also had twice the number of indels than those of *rbcL* (Table 1). Consequently, *rpl32*-*trnL* was selected to sequence all accessions in our study.

**Table 1:** Sequence statistics for *rbcL* and *rpl32-trnL* region on the subsampling.

Comparison factor	*rbcL*	*rpl32-trnL*
Parsimony-uninformative characters	15	11
Parsimony-informative characters	4	41
Tree length (parsimony, ACCTRAN Optimization)	19	60
Number of indels	5	10

### Phylogenetic relationships

**Fig 2:**
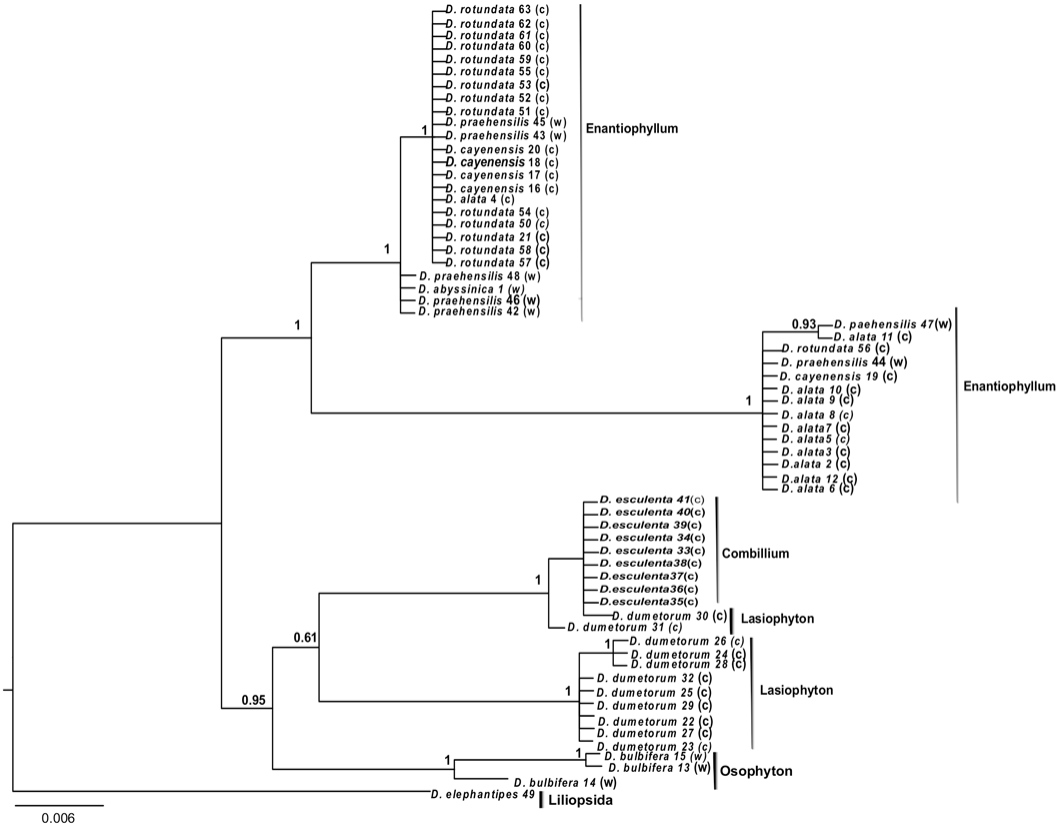
Bayesian phylogenetic tree of wild (w) and cultivated (c) yam accessions with their sections as circumscribed by Knuth (1924).

The Bayesian tree of the *rpl32*-*trnL* region shows expected patterns of congruence with the taxonomic sections of the genus *Dioscorea* (Fig 2), with the exception of section Lasiophyton. Indeed, all sections except Lasiophyton are monophyletic and strongly supported (posterior probability (PP) = 1; Fig 2). The relationships between these sections are also generally well resolved. In contrast to the sections, the species were generally not monophyletic, although it is important also to note that there is little genetic variation within each section. *Dioscorea dumetorum* belongs to Lasiophyton section and has some accessions that are very closely related to *D*. *esculenta* of the section Combilium. *Dioscorea bulbifera*, from section Opsophyton, is the only species that is monophyletic (Fig 2).

### Phylogenetic Diversity and genetic variation partitioning

**Fig 3:**
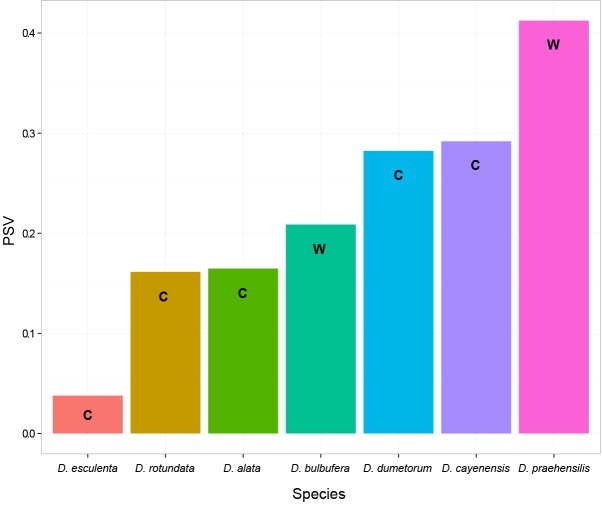
Phylogenetic species diversity (PSV) for each yam species (*Dioscorea* spp.) from Cameroon. The letters c and w indicates the nature of the species with c for cultivated and w for wild.

Phylogenetic diversity estimated for each species can be classified in ascending order from the smallest PD in *D*. *esculenta* to the largest PD obtained in *D*. *praehensilis*. The highest phylogenetic diversity was observed in a wild species, *D*. *praehensilis* (Fig 3). The wild species *D*. *bulbifera* had a PD lower than the cultivated species *D*. *dumetorum* and *D*. *cayenensis*, but it was sampled from a single locality (Ekona). The PD estimated by region showed that the Southwest region had the lowest PD value (PSV = 0.58) and that the Center (PSV = 0.70) and Adamawa (PSV = 0.72) had higher and similar PD values. The partitioning of the total genetic variation informs us on the genetic structure of *Dioscorea* in Cameroon. Together, species and regions explain 60% of the total genetic variation. Ten percent of this variation is co-explained by both species and regions, while individual regions and species fractions explain a significant 4% and 46%, respectively (Table 2). A different result was found when cultivated and wild species were considered separately (Table 2). First, a much larger proportion of the genetic variation could be explained by regions and species for wild species (80%) than for cultivated species (36%). Moreover, there is a fraction of the variation (1%, *p*<0.05) that is explained by regions alone (i.e., after removing the fraction co-explained by species) in wild species, whereas this fraction was of 0% for the cultivated species (Table 2). Together, these results suggest that 1) genetic variation in wild species is more structured than for cultivated species and that 2) intraspecific structure among region was detected for wild species, but not for cultivated species.

**Table 2:** Genetic variation partitioning between species and regions. The adjusted *R* ^*2*^ values and p-values for testable fractions are shown.

Group of species	Species	Regions	Species ∩ Regions	Residual variation
All species	46% (*p* = 0.001)	4% (*p* = 0.28)	10%	40%
Wild species	71% (*p* = 0.001)	1% (*p* = 0.048)	8%	20%
Cultivated species	22% (*p* = 0.012)	0% (*p* = 0.55)	18%	64%

## Discussion

### Phylogenetic relationships and taxonomic implications

Our Bayesian tree supported the monophyly of three out of four *Dioscorea* sections for yams sampled in Cameroon (Enantiophyllum, Combilium, Osophyton). Only section Lasiophyton was found to be paraphyletic due to the grouping of two *D*. *dumetorum* individuals with individuals of section Combilium. Consequently, our results overall supports the taxonomic treatment of Huber [56] based on seed characters, organ morphology and inflorescence development. This author performed a complete taxonomic treatment summarizing the classification systems of Knuth [57] and Bukill [58] by defining sections for the genus *Dioscorea*. The monophyly of the Enantiophyllum section (*D*. *alata*, *D*. *abyssinica*, *D*. *cayenensis*, *D*. *rotundata* and *D*. *praehensilis*) supports the hypothesis of Wilkin et al. [31] that the main Old World lineages of *Dioscorea*, such as the right-twining section Enantiophyllum, are monophyletic. The presence of both wild (*D*. *abyssinica*, *D*. *praehensilis*) and cultivated (*D*. *alata*, *D*. *cayenensis*, *D*. *rotundata*) species within section Enantiophyllum and the proximity between wild and cultivated species supports the idea that the wild species *D*. *abyssinica* and *D*. *praehensilis* could have been involved in the domestication of cultivated species *D*. *cayenensis* and *D*. *rotundata* [59, 60]. Our results also give moderate support for the placement of section Lasiophyllum as sister to *D*. *esculenta* (Sect. Combilium). This pattern is in contradiction with previous studies that placed *D*. *dumetorum* as sister to *D*. *bulbifera* of the section Opsophyton [31,61]. Further studies are clearly required to clarify all the phylogenetic relationships of *D*. *dumetotum*.

In contrast with previous phylogenetic studies on *Dioscorea*, we included eleven accessions of *D*. *alata*, five of *D*. *cayenensis*, *nine* of *D*. *esculenta*, eleven of *D*. *dumetorum*, fifteen of *D*. *rotundata*, three of *D*. *bulbifera*, and seven of *D*. *praehensilis*, which allowed testing hypothesis of species monophyly. This ended up being important as it highlighted the non-monophyly of most species: *D*. *dumetorum*, *D*. *alata*, *D*. *cayenensis*, *D*. *rotundata* and *D*. *praehensilis*. This is even more striking given that a chloroplast gene was used and its lower effective population size compared to that of nuclear loci reduces the likelihood of incomplete lineage sorting. These results will have to be confirmed with other independently evolving markers, but they clearly highlight the need for further investigation of species boundaries in *Dioscorea*.

### Phylogenetic diversity and implications for conservation

Farmers have a strong influence on biological organization in agricultural systems through fragmentation, modification of natural ecosystems, global mixing of species, and breeding programs [62]. This predicts that wild and cultivated species are expected to show different genetic structure, which is what we observed in our results. We found that the genetic diversity was significantly structured by both species and regions for the species studied. The strong taxonomic structure is not surprising as even though not all species were monophyletic, all accessions grouped according to the taxonomic sections of Huber [56]. The genetic structuring by geographical regions was also expected as it reflects a scenario of isolation by distance. However, this regional structure was found to be different between cultivated and wild species. Indeed, for cultivated species, all the regional variation is caused by the fact that different species are found in different regions, whereas intraspecific structure was detected between regions in wild species. The genetic variation was also much more structured by both species and regions for wild than for cultivated species. This different pattern in regional genetic structure between cultivated and wild species might result from the management of the yam germplasm by farmers. Indeed, exchange of yams tubers by farmers among villages could have resulted in a stronger homogenization of the genetic variation among regions for cultivated species compared to wild ones. Although more thorough studies are needed to test this hypothesis, these results nevertheless suggest that different structuring forces affect cultivated and wild species.

Phylogenetic diversity estimates also provided important information on the genetic diversity of wild and cultivated species of yams in Cameroon. We found that the wild species *D*. *praehensilis* has the largest PD among the species studied. This species has often been involved in domestication processes in Cameroon [23] and our results based on chloroplast variation suggest it has a large genetic pool that could potentially offer material for crop improvement.

Previous investigations on *Dioscorea* in Benin have shown that domestication increases the variability within populations [60, 63]. This is likely due to the farming practices in West Africa. Indeed, the farmers often collect wild species in the bushes (Forests, savannah and gallery forest or ancient fallows), especially *D*. *praehensilis*, and then cultivate these wild tubers next to of the cultivated species in the fields. This practice favors the introgression of characters from wild species into cultivated ones. According to Mignouma and Dansi [59], species collected by farmers in bushes can be of different nature (related wild species, interspecific hybrids between wild relatives or between wild species and cultivars) but they are susceptible to influence the genetic variation in a population. These practices likely explain the high PD observed for some cultivated species such as *D*. *cayenensis* and *D*. *dumetorum*.

Finally, some species were found to have a low PD, indicating that they might deserve more specific attention. It is the case of *D*. *esculenta* that is endemic to the Center region and some localities of Mbam, namely ombessa, kédia, Balom and Djanti in Cameroon [23]. *Dioscorea esculenta* is underutilized and not well known by farmers. In our study, it presented the lowest level of PD among the species studied. This could be related to rarity of the species and low effective population sizes, which is directly related to phylogenetic diversity [64]. However, our study is based on a limited geographic sampling and on a chloroplast marker. Therefore, we cannot conclude that *D*. *esculenta* is threatened or endangered such as the edible yam *D*. *bako* from western Madagascar and *D*. *sphaeroidea* from southeastern Brazil [65,66,67]. Nevertheless, further studies would be important to properly assess the threats on this species and determine its conservation status according to the IUCN criteria.

These results on genetic diversity are based on a single chloroplast marker and they will certainly need to be confirmed with additional unlinked markers from the nuclear genome. Because genetic variation at each locus (and for each organelle) represents a unique outcome of the stochastic population genetic processes, it might not reflect the overall genomic structure. Moreover, the uniparental maternal transmission of chloroplasts [63] reduces its effective population size by one half compared to nuclear markers, which results in stronger genetic drift in population [68, 69,70] and stronger population structure [71]. But despite these shortcomings, the present results have the advantage of comparing all species at the same level despite their variation in ploidy, and it provides a good approach to identify where future genetic investigations should spend their efforts.

Our results showed that PD is very useful to get a broad picture of the genetic structure and diversity in a group of related organisms in a territory. Phylogenetic diversity provides intraspecific genetic diversity information as well as knowledge on the phylogenetic relationships among species. The application of the phylogenetic diversity in the genus *Dioscorea* from Cameroon helped to clarify the relationships among the species, a very important aspect to properly understand the structure of genetic variation in yams, but more importantly highlighted that species boundaries in *Dioscorea* deserve more attention. Moreover, our results highlighted species with low PD that deserves to be studied in more detail. For instance, the use of reduced genome sequencing approaches (RADseq and GBS) would allow obtaining both stronger phylogenetic hypotheses and more precise estimates of genetic diversity and population genetic structure. Nonetheless, the implementation of phylogenetic diversity tools in the genus *Dioscorea* from Cameroun represents a very useful first step toward the establishment of a conservation program for yams. Phylogenetic diversity has been repeatedly suggested to be one of the best strategies for the preservation of genetic resources because it can be related to processes such as extinction [3], biotic variation [72], ecosystem functioning [73], and even ecosystem services [74]. Our study suggests that the Phylogenetic Diversity toolbox can be extended to infer genetic structure in non-model crops that consist of several closely related species such as yams.
